# Positive-strand RNA virus replication organelles at a glance

**DOI:** 10.1242/jcs.262164

**Published:** 2024-09-10

**Authors:** Viktoriya G. Stancheva, Sumana Sanyal

**Affiliations:** Sir William Dunn School of Pathology, University of Oxford, South Parks Road, Oxford, OX1 3RE, UK

**Keywords:** RNA virus, Replication organelles, Virus–host interaction

## Abstract

Membrane-bound replication organelles (ROs) are a unifying feature among diverse positive-strand RNA viruses. These compartments, formed as alterations of various host organelles, provide a protective niche for viral genome replication. Some ROs are characterised by a membrane-spanning pore formed by viral proteins. The RO membrane separates the interior from immune sensors in the cytoplasm. Recent advances in imaging techniques have revealed striking diversity in RO morphology and origin across virus families. Nevertheless, ROs share core features such as interactions with host proteins for their biogenesis and for lipid and energy transfer. The restructuring of host membranes for RO biogenesis and maintenance requires coordinated action of viral and host factors, including membrane-bending proteins, lipid-modifying enzymes and tethers for interorganellar contacts. In this Cell Science at a Glance article and the accompanying poster, we highlight ROs as a universal feature of positive-strand RNA viruses reliant on virus–host interplay, and we discuss ROs in the context of extensive research focusing on their potential as promising targets for antiviral therapies and their role as models for understanding fundamental principles of cell biology.

## Teaser

**Summary:** Positive-strand RNA viruses induce diverse replication organelles; understanding their biogenesis provides insight into viral replication and opens avenues for developing broad-spectrum antiviral agents.

## Introduction

Positive-sense (+) single-stranded RNA (+ssRNA) viruses, also known as positive-strand RNA viruses, comprise a diverse group that includes members such as dengue virus (DENV), hepatitis C virus (HCV) and SARS-CoV-2. These viruses share a common replication strategy: the formation of specialised compartments termed replication organelles (ROs) (Belov and van Kuppeveld, 2012; Den Boon and Ahlquist, 2010). ROs are virus-induced structures that serve as factories for viral RNA synthesis, providing a conducive environment for assembly and function of the replication complex. This Cell Science at a Glance article focuses on membrane-derived ROs of +ssRNA viruses, distinct from the membrane-less replication factories formed by negative-strand RNA viruses (Hoenen et al., 2012; Nevers et al., 2020).

**Figure:**
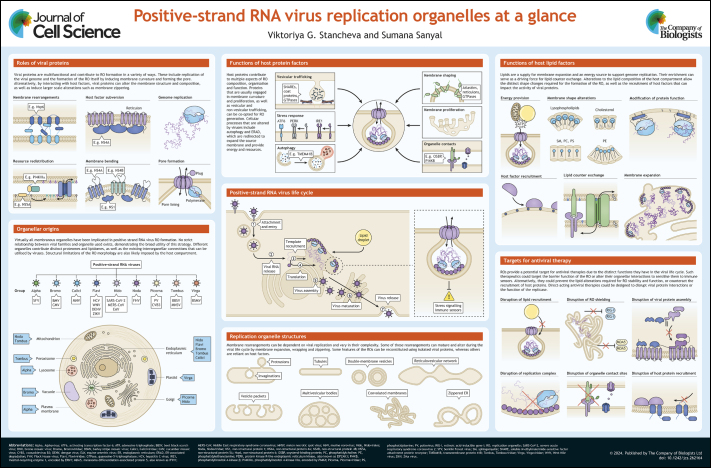
See Supplementary information for a high-resolution version of the poster.

ROs of +ssRNA viruses arise from the remarkable ability of viruses to reshape host cell membranes, including the endoplasmic reticulum (ER), Golgi, endosomes, mitochondria and plasma membrane (Nguyen-Dinh and Herker, 2021). Although the specific morphology and biogenesis of ROs varies among virus families (see poster), ROs can be broadly grouped into invagination and protrusion morphotypes, the latter being an intermediate state of double-membrane vesicles (DMVs) (Nguyen-Dinh and Herker, 2021; Wolff et al., 2020b). In general, protrusion-type ROs tend to derive more from the secretory pathway compared to the wider range of organelles generating invagination-type ROs. The source of ROs can vary even within virus families and cell types, as exemplified by the Tombusviridae family (Xu and Nagy, 2014) and coxsackievirus B (CVB) (Melia et al., 2019; Park et al., 2021).

A feature of many ROs is a membrane-spanning pore, recently termed the ‘replicopore’ (Zimmermann et al., 2023), that acts as a controlled gateway between the organelle interior and cytoplasm, regulating the import of essential replication components and the export of newly synthesised viral RNA (Ertel et al., 2017; Wolff et al., 2020a). However, such pores have not been demonstrated for all +ssRNA viruses; for example, picornaviruses use protrusion-like organelles as anchoring sites for replication complexes (Richards and Jackson, 2012). Invagination-type ROs are typically 40–90 nm indentations connected to the cytoplasm via the pore (Kopek et al., 2007), and these ROs can be subdivided based on reliance on polymerase activity. The size of Semliki Forest virus (SFV) ROs correlates with genome length (Kallio et al., 2013), likely following the ‘synthesis model’, where the entire RNA acts as a structural component and its incorporation into the spherule during replication determines the final spherule size. Orthoflaviviruses and brome mosaic virus (BMV) form polymerase-independent invaginations (Schwartz et al., 2004; Welsch et al., 2009). Meanwhile, protrusion-type ROs display more variability, maturing from single- to double- or multi-membrane vesicles or tubules (Knoops et al., 2008; Romero-Brey et al., 2012). This structural diversity can vary throughout infection, as exemplified by coronavirus DMVs, which range from 100 nm to 400 nm in size (Snijder et al., 2020; Wolff et al., 2020a).

The formation and maintenance of ROs requires a delicate interplay between viral and host factors, with non-structural proteins, replicase components, membrane-shaping proteins, lipid enzymes and trafficking components being crucial (Nagy and Pogany, 2012). Viruses actively manipulate host metabolism to supply lipids for membrane proliferation. Fatty acids derived from lipid droplet turnover undergo β-oxidation to support the energy demands of replication (Heaton and Randall, 2011; Zhang et al., 2018). Viruses also recruit host lipid biosynthetic enzymes, exploit transport pathways and establish close contacts between ROs and subcellular organelles (Barajas et al., 2014). RO compartmentalisation also serves to evade host antiviral responses by minimising viral RNA exposure to cytoplasmic sensors and nucleases (Van Hemert et al., 2008a,b). However, host cells have counter-evolved strategies to detect and target ROs, leading to an ongoing evolutionary arms race (Schoggins, 2014). This Cell Science at a Glance article and the accompanying poster provide an overview of the diverse morphologies and origins of ROs across different +ssRNA virus families, highlighting the importance of virus–host interactions that drive RO biogenesis and function, as well as the methods used to study viral ROs (see Box 1).

**Box 1. Methods to study +ssRNA virus ROs**

The advancement of technical approaches has been instrumental in addressing morphological and functional aspects of ROs. Electron microscopy (EM) and electron tomography (ET) have brought the study of ROs to the forefront (Den Boon et al., 2024), with *in situ* cryo-EM allowing the analysis of proteins and membranes in a near-native environment (Klein et al., 2020). Although primarily a structural approach, ET has been essential in delineating replication-dependent (Welsch et al., 2009) and replication-independent ROs (Goellner et al., 2020). *In situ* cryo-ET, combined with subtomogram averaging, can deliver the structure of pore complexes and their minimal components, as well as be used with genetic alterations for correlative studies. Super-resolution fluorescence microscopy has also provided key evidence regarding the composition, structure and assembly of ROs (Andronov et al., 2024), whereas metabolic labelling has indicated the stability of replication complexes in cells (Lan et al., 2023). Several biochemical techniques have been developed to address specific mechanistic details. Lipid contributions to the ROs can be analysed by using lipid probes with microscopy and mass spectrometry (Schultz et al., 2022), whereas contact sites between organelles that support RO formation can be studied using split TurboID (Cho et al., 2020). *In vitro* reconstitution of RO-like structures on giant unilamellar vesicles can demonstrate the minimal components of these structures (Kovalev et al., 2020). Model viruses with minimalistic genomes, such as Flock House virus (FHV), and replication-independent expression systems (Goellner et al., 2020) can be used to interrogate the contribution of specific components in a simplified setup. Lower eukaryotes such as yeast (as an infection model) have driven the understanding of different aspects of RO formation (Nagy et al., 2014). Importantly, multiple approaches – including metabolic labelling, structural studies and replication-independent systems – can be used in combination to provide mechanistic insights into processes such as the relation of ROs and RNA synthesis. Lastly, mathematical modelling can predict the outcome of events such as drug treatment or mutations (Sunagawa et al., 2023), relying on the similarities between replication kinetics of different viruses and a detailed understanding of all components in the system.

## Viral proteins involved in RO biogenesis

The viral proteins that drive RO biogenesis can be broadly categorised based on their function, with the non-structural proteins being essential (see poster). These are multifunctional proteins that possess various enzymatic activities, such as RNA-dependent RNA polymerase (RdRp), helicase and protease functions, all of which are crucial for viral genome replication (Chen et al., 2020a; Rathnayake et al., 2020; Shiryaev et al., 2023). Others, such as the NS4A, NS4B and NS1 proteins of orthoflaviviruses, interact with host membranes to induce membrane curvature and promote formation of invaginations (Akey et al., 2014; Ci et al., 2020). Similarly, in coronaviruses such as SARS-CoV-1, SARS-CoV-2 and MERS-CoV, Nsp3 and Nsp4 are sufficient to induce formation of DMVs that serve as the primary sites of viral replication, and Nsp6 induces formation of zippered ER membranes connecting DMVs to the ER, facilitating lipid flux (Oudshoorn et al., 2017; Zimmermann et al., 2023).

Studies on the picornaviruses poliovirus, coxsackievirus B3 (CVB3) and encephalomyocarditis virus (EMCV) have provided insights into picornavirus ROs, which are predominantly DMVs. In studies of poliovirus, viral proteins 2BC and 3A have been found to be sufficient to induce DMVs that resemble those formed during infection, likely deriving from the ER but excluding ER-resident proteins (Suhy et al., 2000). For CVB3, the transformation from single-membrane structures to DMVs occurs via membrane pairing and enwrapping, with viral RNA synthesis linked to DMV formation (Limpens et al., 2011). EMCV also forms single-membrane ROs early in infection that transform into DMVs, requiring cellular acidification after a ‘transition point’, separating RNA replication from virion maturation (Galitska et al., 2023; Melia et al., 2018). The specific mechanisms inducing these structures remain to be fully elucidated; however, studies on poliovirus 2C and 3A (Suhy et al., 2000), 2BC-triggered lipidation of LC3 (MAP1LC3) proteins (Dahmane et al., 2022), and EMCV 3A (Galitska et al., 2023), suggest that membrane-associated viral replication proteins play key roles in generating ROs.

Another group of viral proteins essential for RO biogenesis are those that form the pore complex, which typically includes membrane-interacting proteins lining the pore, potential subunits observed as a plug at the opening and the viral polymerase (Ertel et al., 2017; Wolff et al., 2020a). For example, a narrow neck (∼10 nm in diameter) of unknown molecular composition connects DENV ROs with the cytosol (Welsch et al., 2009), whereas in coronaviruses, the Nsp3 protein forms the crown of the pore (Wolff et al., 2020a). Interestingly, pore components are not exclusively non-structural proteins, as the nucleocapsid has been shown to associate with the pore complex in some cases, such as in SARS-CoV-2, binding to Nsp3 at the crown of the pore and likely allowing the delivery of viral RNA from the vesicle interior to an assembling virion (Scherer et al., 2022). However, the precise function of the pore remains an open question that warrants further investigation.

Viral proteins also play an important role in recruiting and manipulating host factors necessary for RO formation by mimicking or hijacking host proteins involved in membrane trafficking, lipid metabolism and organelle dynamics (Nagy and Pogany, 2012). For instance, the poliovirus 3A protein interacts with the host protein GBF1, a guanine-nucleotide-exchange factor involved in membrane trafficking, to facilitate the formation of ROs. Similarly, HCV NS5A interacts with phosphatidylinositol 4-kinase IIIα (PI4KIIIα, encoded by *PI4KA*), a lipid kinase, to promote synthesis of phosphatidylinositol 4-phosphate (PI4P), which is essential for the formation and maintenance of ROs (Reiss et al., 2011). Another example is the interaction between the West Nile virus (WNV) NS4A protein and reticulon 3.1A (encoded by *RTN3*), which induces membrane curvature to facilitate RO formation (see section ‘Host proteins involved in RO biogenesis’ below; Aktepe et al., 2017). By interacting with these host factors, viral proteins redirect cellular resources towards the construction of ROs.

## Host proteins involved in RO biogenesis

The formation and function of ROs heavily depends on the recruitment and manipulation of host factors. These proteins are involved in membrane remodelling, lipid metabolism and the establishment of favourable microenvironments for viral replication (see poster) (Lan et al., 2023; Williams et al., 2023). Viruses often hijack host proteins that naturally participate in inducing membrane curvature, such as reticulons and other ER morphogens including atlastins, receptor expression-enhancing proteins (REEPs) and ARF GTPases (Li et al., 2023b; Neufeldt et al., 2019). For example, enteroviruses subvert reticulons to curve ER membranes into vesicle-connected replication tubules (Melia et al., 2018; Tang et al., 2007), and coronavirus replication has been shown to rely on both reticulons (Williams et al., 2023) and ER sheets (Cortese et al., 2020). Flaviviruses recruit reticulons, atlastins and other GTPases to sites of RO formation, leveraging their membrane-bending properties to create the characteristic RO structures (Aktepe et al., 2017; Neufeldt et al., 2019). Similarly, tombusviruses co-opt ER-localised soluble *N*-ethylmaleimide-sensitive factor attachment protein receptor (SNARE) proteins (Sasvari et al., 2018), which mediate vesicle fusion and are essential for membrane trafficking and remodelling (Jahn et al., 2024).

Many viruses hijack the host secretory pathway (Hsu et al., 2010), so host proteins involved in vesicle trafficking and organelle dynamics also play key roles in RO biogenesis. This includes coat protein complex II (COPII), which facilitates transport of viral and host components to the RO; for example, poliovirus recruits COPII components SEC13 and SEC31 to sites of RO formation (Trahey et al., 2012). Furthermore, viruses induce restructuring of membrane contact sites, allowing non-vesicular transport of metabolites and signalling molecules between subcellular organelles and ROs (Strating and Van Kuppeveld, 2017). Tombusviruses co-opt ER–chloroplast and ER–peroxisome tethering complexes to supply lipids and energy (Barajas et al., 2014; Sasvari et al., 2018), whereas enteroviruses manipulate ER–Golgi membrane contact sites [via oxysterol-binding protein (OSBP) and PI4KB] to direct membrane flow (McPhail et al., 2020; Melia et al., 2019). In HCV, the NS3/4A protease complex cleaves mitochondrial antiviral signalling protein (MAVS) from ER–mitochondria contacts, but not from mitochondria, to disrupt retinoic acid-inducible gene I (RIG-I) signalling, which forms part of the innate immune response (Horner et al., 2011; Piccoli et al., 2009).

In addition, viruses often manipulate cellular processes to their advantage, such as autophagy (Li et al., 2020; Teo et al., 2021; Wong and Sanyal, 2020) and the unfolded protein response (UPR), which is a cellular stress response pathway initiated when the ER becomes overloaded with unfolded or misfolded proteins. Viruses can also target ER-associated degradation (ERAD), which recognises and degrades misfolded proteins at proteasomes as part of the ER quality control mechanisms. Here, flaviviruses activate inositol-requiring enzyme 1 (IRE1, encoded by *ERN1*) and activating transcription factor 6 (ATF6) UPR sensors to expand the ER while suppressing inflammatory signalling via proteasomal degradation of immune components (Ambrose and Mackenzie, 2013; Yu et al., 2006). Flaviviruses also induce lipophagy (the autophagic degradation of lipid droplets) while suppressing ER-phagy (degradation of the ER) to maintain stable ROs (Lan et al., 2023; Zhang et al., 2018). Meanwhile, coronavirus DMVs require non-lipidated LC3 proteins (a marker of autophagy), independent of the autophagy machinery (Reggiori et al., 2010).

Finally, the ER transmembrane protein TMEM41B has emerged as a key host factor in the replication of diverse +ssRNA viruses (Ji et al., 2022; Schneider et al., 2021). TMEM41B is involved in autophagosome formation and lipid mobilisation (Moretti et al., 2018). Although the exact mechanisms remain to be elucidated, TMEM41B might contribute to RO formation by regulating lipid flux and membrane remodelling (Huang et al., 2021) via its scramblase activity, facilitating membrane fluidity and curvature necessary for RO formation.

## The role of host lipids in RO biogenesis

Like host proteins, host lipids are also essential components in the formation and function of ROs. Viruses actively manipulate host metabolism to ensure a steady supply of specific lipids required for membrane proliferation and to support the unique structural and energetic demands of viral replication (Heaton and Randall, 2011; Pombo and Sanyal, 2018). The lipid composition of ROs can be distinct from that of the host cell membranes from which they originate (summarised in Zhang et al., 2019), highlighting the importance of virus-directed lipid remodelling in RO biogenesis (see poster).

ROs form as an extension of host membrane-bound organelles but impose unique topological requirements, which are anticipated to be satisfied by a distinct lipid makeup that affects the physical properties of membranes and their protein composition. Membranes of both RO morphotypes are enriched in sterols, which contribute to membrane rigidity and curvature, allowing optimal RO environments for virus replication (Mackenzie et al., 2007; Roulin et al., 2014). Sterols can also impact the activity of viral proteins, such as the enterovirus 3CD^pro^ protein, which is required for formation of the replication complex. Cholesterol organisation and abundance within ROs has been found to be crucial for 3CD^pro^ processing kinetics (Ilnytska et al., 2013).

Glycerophospholipids, the main structural components of cellular membranes, also play important roles; they can impact membrane curvature and protein recruitment based on molecular shape, with cone-shaped lipids, such as phosphatidylethanolamine (PE) and phosphatidic acid, promoting negative curvature, and inverted cone-shaped lipids, such as lysophosphatidylcholine, promoting positive curvature (Chen et al., 2018). These properties are exploited to facilitate RO formation and function. For example, several +ssRNA viruses promote accumulation of phosphatidylcholine at ROs via localised synthesis (Zhang et al., 2016). PE, which is enriched in some ROs, affects tomato bushy stunt virus (TBSV) replication by facilitating viral protein enrichment and organisation (Xu and Nagy, 2016).

Phosphatidylinositols, although not abundant, can play significant roles in RO formation and function. PI4P is used by various +ssRNA viruses to recruit proteins and lipids to ROs, often via the lipid transport protein OSBP, which drives the counter exchange of PI4P for cholesterol (Arita, 2014; Mesmin et al., 2013). This mechanism is important for RO formation in viruses such as rhinoviruses (Roulin et al., 2014) and HCV (Wang et al., 2014).

Sphingolipids have been implicated in the replication of some +ssRNA viruses but have not been directly localised to ROs. Their utilisation can vary between viruses of the same genus. For example, WNV infection is enhanced by ceramide accumulation, whereas DENV replication is inhibited by ceramide (Aktepe et al., 2015; Martín-Acebes et al., 2014), highlighting the complex nature of virus–host lipid interactions in RO function.

Viruses also manipulate the distribution and composition of lipids within RO membranes by exploiting lipid transfer proteins and transporters to shuttle lipids between organelles and ROs (Hsu et al., 2010). For example, OSBPs are hijacked by various viruses, including picornaviruses and HCV, to facilitate the delivery of cholesterol to ROs (Arita et al., 2013; Barajas et al., 2014; Roulin et al., 2014; Wang et al., 2014). In picornavirus infection, OSBP is recruited to RO membranes, where it mediates the exchange of PI4P for cholesterol, leading to an enrichment of cholesterol in the RO (Roulin et al., 2014). Similarly, HCV exploits OSBP to create a PI4P gradient that drives the accumulation of cholesterol in the ROs (Wang et al., 2014). HCV also hijacks four-phosphate adaptor protein 2 (FAPP2, also known as PLEKHA8) to facilitate the transfer of glycosphingolipids to ROs, likely promoting membrane curvature and stability (Khan et al., 2014).

Accordingly, viruses modulate host lipid metabolism to meet the energy demands of viral replication (Diamond et al., 2010). The synthesis of new viral RNA and proteins requires a significant amount of energy, which can be derived from fatty acid oxidation (Heaton and Randall, 2010). For example, DENV has been shown to upregulate the expression of genes involved in fatty acid synthesis, ensuring a steady supply of ATP for replication (Heaton et al., 2010). An increase in fatty acid oxidation is accompanied by a corresponding decrease in lipid storage, as evidenced by the depletion of lipid droplets in DENV-infected cells (Heaton and Randall, 2010; Zhang et al., 2018). Similarly, HCV has been found to enhance the expression of genes involved in lipid catabolism, particularly those related to mitochondrial and peroxisomal fatty acid oxidation (Diamond et al., 2010). This metabolic reprogramming is essential for HCV replication, as treatment with saturated and mono-unsaturated fatty acids significantly increases viral RNA levels and protein expression (Kapadia and Chisari, 2005). As our knowledge of the lipid requirements and remodelling events that occur during viral replication continues to grow, it will be important to explore strategies for disrupting these virus–host lipid interactions as a means of inhibiting viral replication and preventing disease (see Box 2).

**Box 2. ROs as a target for antiviral therapy**

ROs present two main approaches for the development of antiviral therapies: targeting of viral components and targeting of host factors (see poster) (Li et al., 2023a). The targeting of host factors has the benefit of reduced sensitivity to viral adaptations and the possibility of affecting a variety of unrelated viruses if a shared host factor is central to their RO biology. Alternatively, direct-acting antiviral agents have proven successful in disrupting viral proteins involved in RO formation.

One example of a direct-acting antiviral treatment is a drug developed by Janssen, currently in phase II clinical trials, that targets the early association of DENV NS4B with NS3, a step needed for RO establishment (Goethals et al., 2023). Similarly, daclatasvir, which is directed at HCV NS5A, is used as an antiviral agent against HCV and impacts biogenesis of ROs independent of RNA replication (Berger et al., 2014; Nelson et al., 2015). It has been proposed to target domain I of NS5A, potentially affecting NS5A dimerisation and interaction with cyclophilin A (also known as PPIA). Importantly, inhibitors of cyclophilin A also act on MERS-CoV, highlighting the potential for transferable approaches to treat viruses with similar RO morphotypes (De Wilde et al., 2011).

Another direction is to target the lipid composition of ROs. This is demonstrated by the US Food and Drug Administration-approved FASN inhibitor, which reduces lung pathology caused by SARS-CoV-2 by inhibiting fatty acid and palmitoylated protein synthesis (Chu et al., 2021b). Inhibition of PI4KB has potential for broad-spectrum effects but is currently associated with high toxicity and development of resistance (Mejdrová et al., 2017). However, this remains a promising approach, as the downstream factor OSBP has been targeted with less associated cytotoxicity (Wang et al., 2014). Upstream modulation of RO cholesterol enrichment has been targeted by inhibition of sterol regulatory element-binding protein (SREBP), which has been shown to lead to increased survival of MERS-infected mice (Yuan et al., 2019). Meanwhile, the cholesterol-lowering drug lovastatin has been shown to inhibit the replication of several viruses, including HCV and DENV, although its effect depends on the cell system used (Boldescu et al., 2017). Collectively, these results suggest that altering lipid fluxes is a feasible avenue for future research.

## ROs at the interface of host immunity and disease

ROs play a crucial role in shielding viral RNA from detection by cytoplasmic pattern recognition receptors such as RIG-I and MDA5 (also known as IFIH1), which are essential for activating innate immune responses, including the production of type I interferons (IFNs) and proinflammatory cytokines (Chow et al., 2018). For example, the HCV membranous web acts as a barrier that excludes RIG-I and MDA5, preventing sensing of the viral genome (Neufeldt et al., 2016). Similarly, the convoluted membranes of DENV have been shown to alter mitochondrial morphology and immune activation by MAVS at nascent ER–mitochondria sites (Chatel-Chaix et al., 2016). Other observations supporting the role of ROs in immune evasion include the sensitising effect of disrupting RO formation and the correlation of IFN induction with leakage of viral RNAs outside of the ROs (Scutigliani and Kikkert, 2017). For instance, treatment with an inhibitor of the yellow fever virus (YFV) NS4B protein not only blocks RO formation, but also induces a robust RIG-I-dependent IFN response, highlighting the importance of intact ROs in evading innate immune sensing (Gao et al., 2022). It is worth noting that enteroviruses can replicate in the absence of ROs, albeit at an altered location, and delayed RO formation under PI4KB inhibition does not lead to enhanced innate immune activation (Melia et al., 2017). Furthermore, +ssRNA viruses have multiple other means of counteracting innate immune sensing of viral RNAs, suggesting that they could be capable of propagating in the context of dysfunctional ROs.

From the host perspective, ROs are targets of immune responses. IFN-stimulated gene (ISG) products – such as IFI6, which acts at the ER to prevent flavivirus RO formation (Richardson et al., 2018), and cholesterol 25-hydroxylase, which depletes membrane cholesterol (Wang et al., 2020) – are examples of host factors that target ROs. IFN treatment has been shown to limit free cholesterol and vice versa (Teo et al., 2023). Additionally, the ISG protein viperin (also known as RSAD2) localises to the ROs of several viruses, including DENV and HCV, where it disrupts the lipid composition and membrane integrity (Helbig et al., 2013; Wang et al., 2012). ISG15 has been reported to interfere with SARS-CoV-2 replication; however, whether this is via modification of ROs merits further investigation (Fu et al., 2021).

The formation of ROs can trigger the activation of the UPR, which has been linked to the induction of inflammatory responses during viral infection (Zhang and Kaufman, 2008). For example, Zika virus (ZIKV) RO formation activates the IRE1 branch of the UPR, leading to production of type I IFNs and inhibition of viral replication (Tan et al., 2018).

Despite evidence supporting interactions between ROs and the immune response, clear links to the pathology of the associated diseases are not yet established. However, it is possible that ROs can be determinants of host range, as observed in MERS-CoV infection, where conserved mutations in the Nsp6 protein are associated with differences in viral replication efficiency between humans and camels (Dudas et al., 2018; So et al., 2023). Variations in RO formation and function might therefore contribute to the adaptation of viruses to different hosts.

The ER-localised enzyme placental alkaline phosphatase (ALPP) exemplifies how host factors involved in RO formation and function can affect disease presentation. ALPP stabilises the ZIKV replication complex and is primarily expressed in the placenta, potentially contributing to the severe congenital abnormalities associated with ZIKV infection during pregnancy (Chen et al., 2020b). The specific expression of ALPP in the placenta might explain the unique vulnerability of this tissue to ZIKV infection and the associated adverse pregnancy outcomes. In contrast, some proteins and their variants, such as an oligoadenylate synthetase 1 (OAS1) isoform that localises to the endomembrane system, can be protective against severe disease (Soveg et al., 2021).

RO formation can lead to dysregulation of cellular processes, such as apoptosis and autophagy, contributing to tissue damage and disease progression. Coronavirus RO formation can induce apoptosis in infected cells, which might contribute to the severe lung pathology observed in COVID-19 patients (Chu et al., 2021a). Similarly, ZIKV RO formation has been linked to dysregulation of autophagy, potentially contributing to neurological complications (Liang et al., 2016). In HCV infection, RO formation promotes the survival of infected cells by inhibiting apoptosis and promoting evasion of immune-mediated clearance, leading to chronic liver disease, cirrhosis and hepatocellular carcinoma (Bantel and Schulze-Osthoff, 2003). As our knowledge of the interplay between ROs and host immunity expands (Box 2), identifying novel therapeutic targets and developing strategies to harness the host immune system to combat viral infections will be crucial (see poster).

## Conclusions and outstanding questions

In this Cell Science at a Glance article, we have highlighted the diverse morphologies and origins of ROs across different +ssRNA virus families, emphasising the importance of virus–host interactions that drive RO formation and function. Despite significant advances in our understanding of the ultrastructural characteristics of ROs, several key knowledge gaps remain. Challenges include elucidating the precise mechanisms by which viral and host proteins interact to drive membrane remodelling and RO formation, the role of lipid composition and membrane dynamics in RO function and stability, and the specific contributions of membrane contact sites and lipid transfer proteins to RO biogenesis. Additionally, the mechanisms by which ROs evade or modulate host immune responses remain to be characterised. Addressing these knowledge gaps will require development of new experimental tools and model systems (Box 1), as well as collaborative efforts across multiple disciplines. Ultimately, a deeper understanding of RO biogenesis will not only provide fundamental insights into virus–host interactions but also inform the development of novel strategies for antiviral treatments.

## Poster

Poster

## Panel 1. Positive-strand RNA virus life cycle

Panel 1. Positive-strand RNA virus life cycle

## Panel 2. Viral replication organelle origins and structures

Panel 2. Viral replication organelle origins and structures

## Panel 3. Roles of viral proteins

Panel 3. Roles of viral proteins

## Panel 4. Functions of host protein factors

Panel 4. Functions of host protein factors

## Panel 5. Functions of host lipid factors

Panel 5. Functions of host lipid factors

## Panel 6. Targets for antiviral therapy

Panel 6. Targets for antiviral therapy
