# Supplementary figures and images for: Positive-strand RNA virus replication organelles at a glance
Source: J Cell Sci. 2024 Sep 10;137(17):jcs262164. doi: 10.1242/jcs.262164 (PMC11423815; doi:10.1242/jcs.262164)

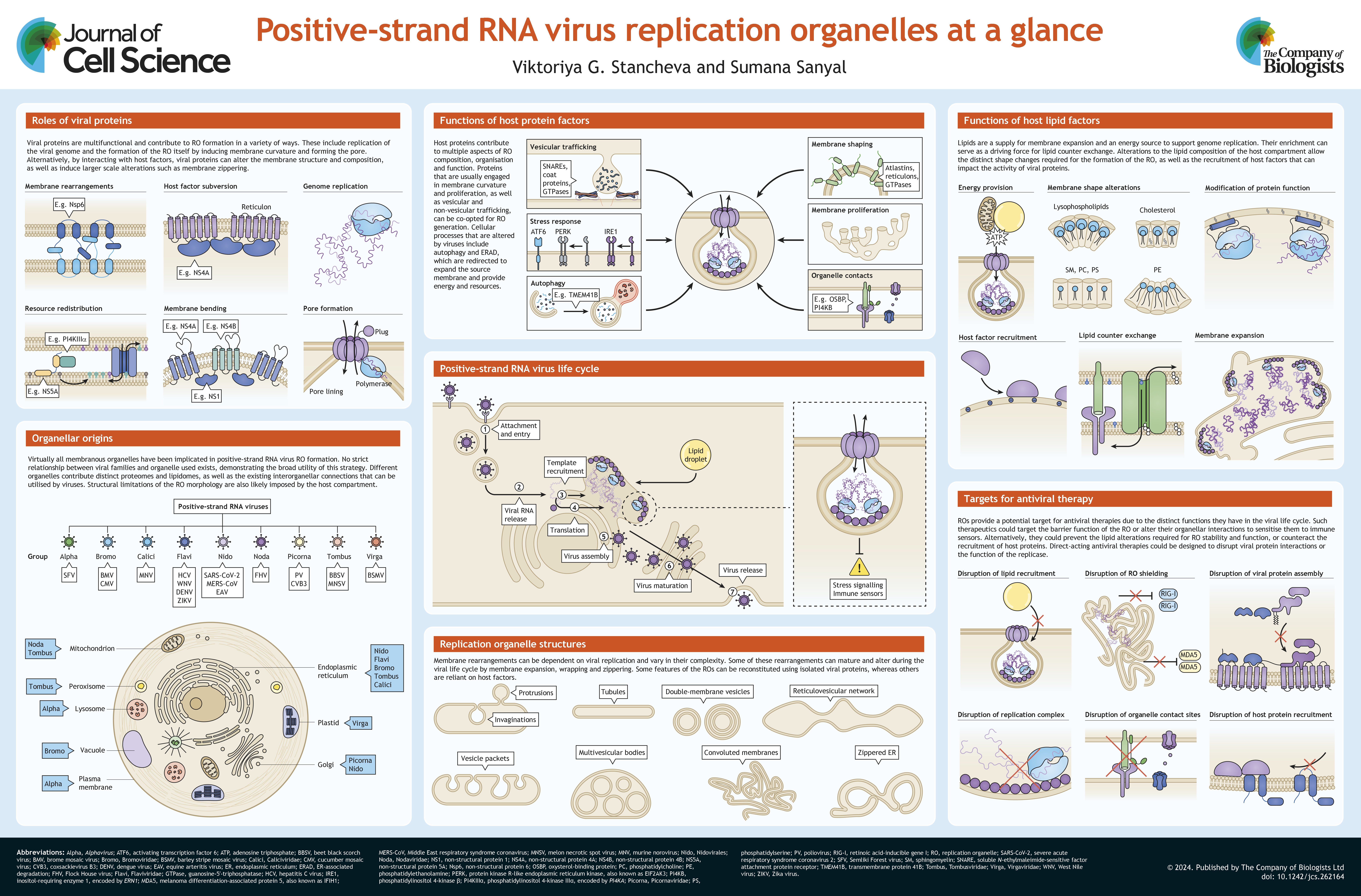

Supplement: Poster [file joces-137-262164-s1.jpg]

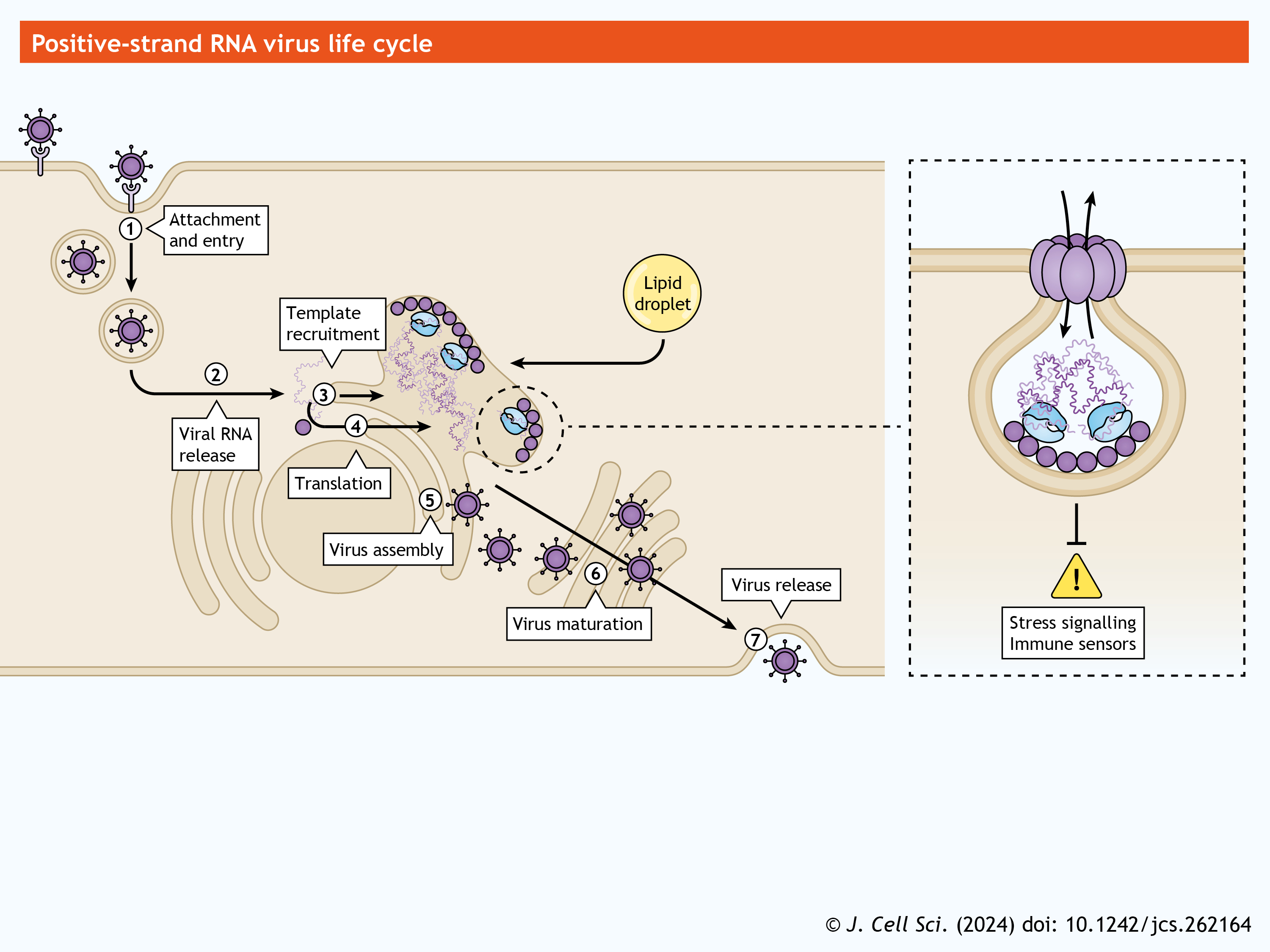

Supplement: Panel 1. Positive-strand RNA virus life cycle [file joces-137-262164-s2.jpg]

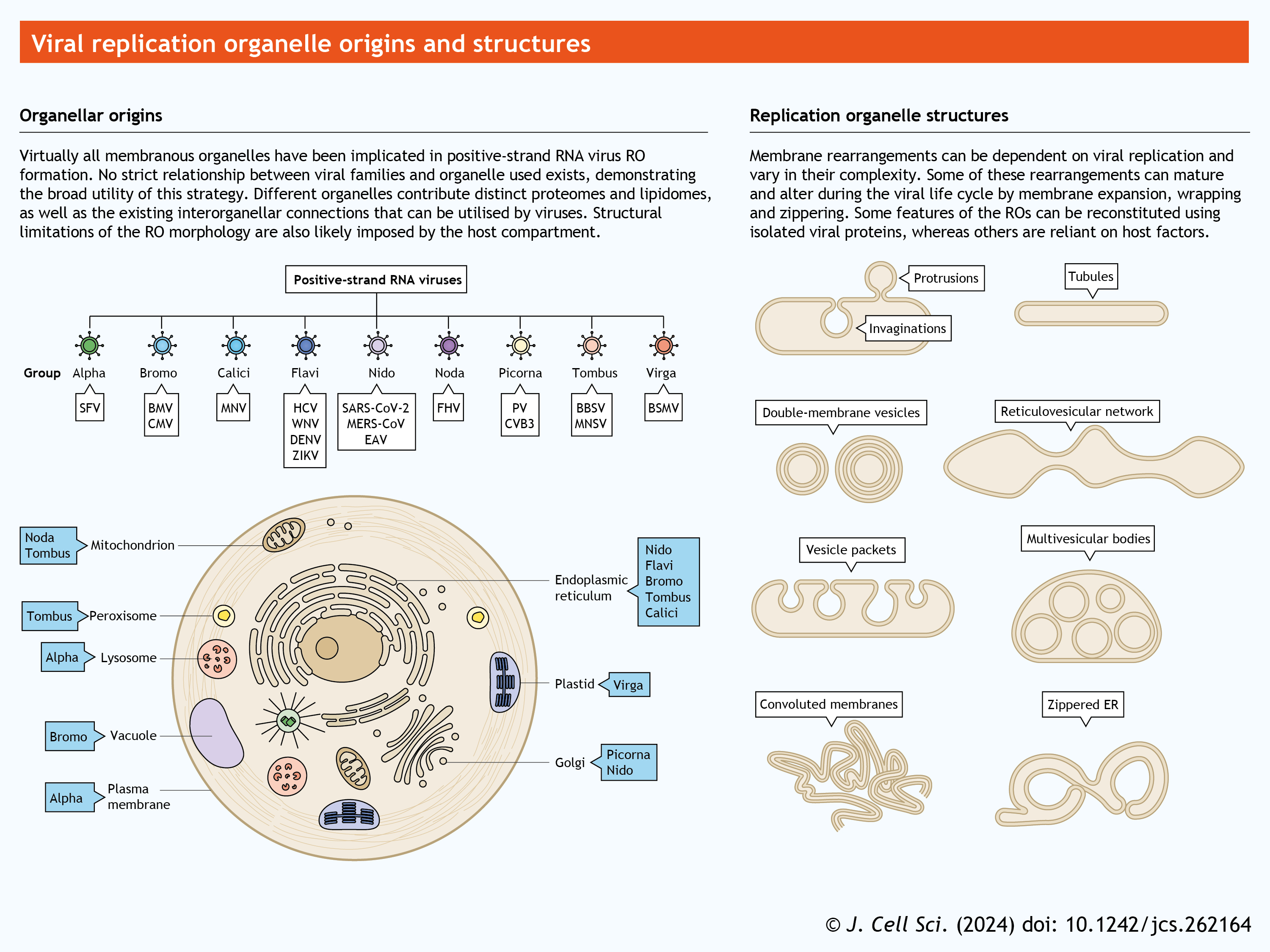

Supplement: Panel 2. Viral replication organelle origins and structures [file joces-137-262164-s3.jpg]

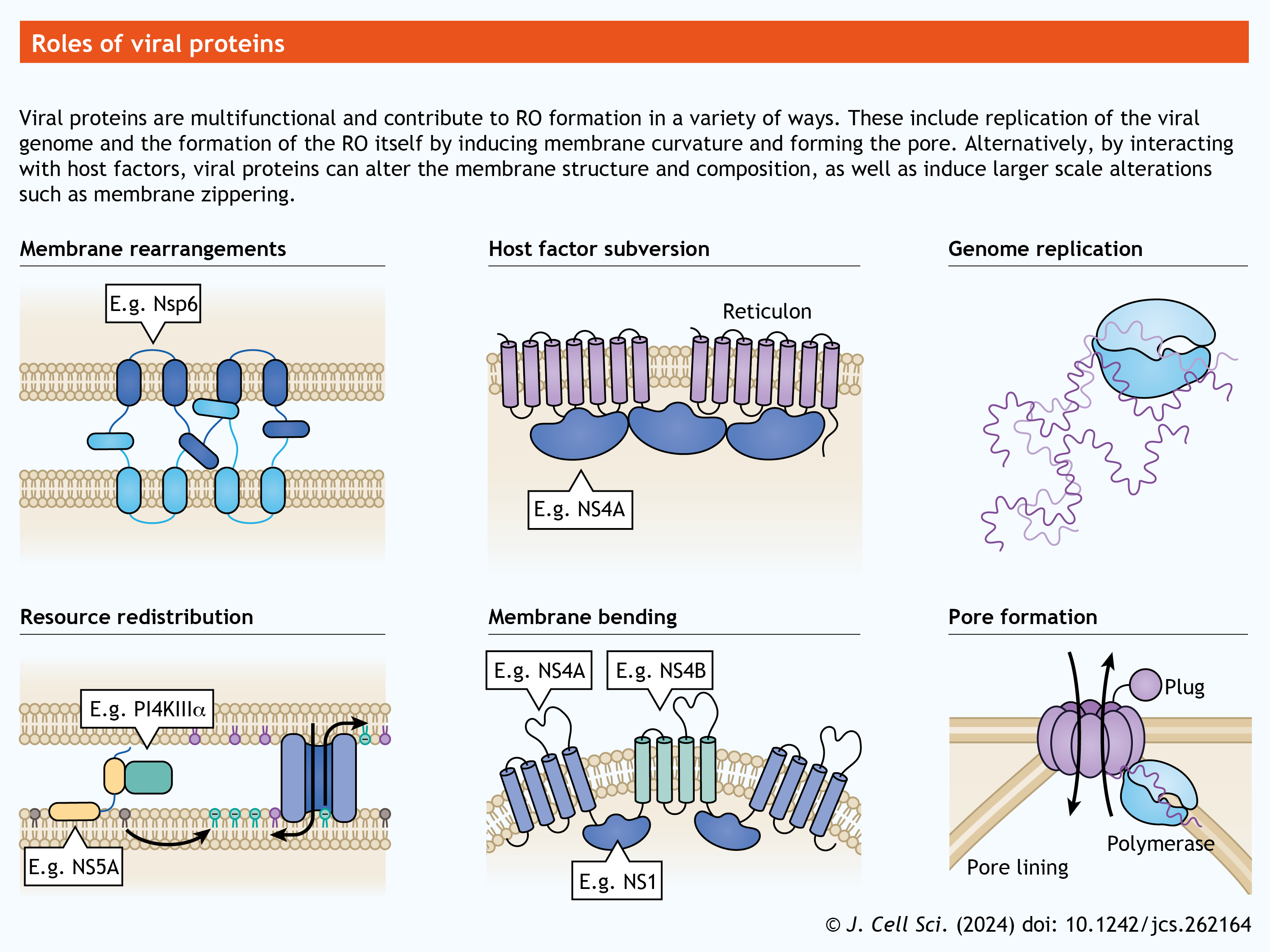

Supplement: Panel 3. Roles of viral proteins [file joces-137-262164-s4.jpg]

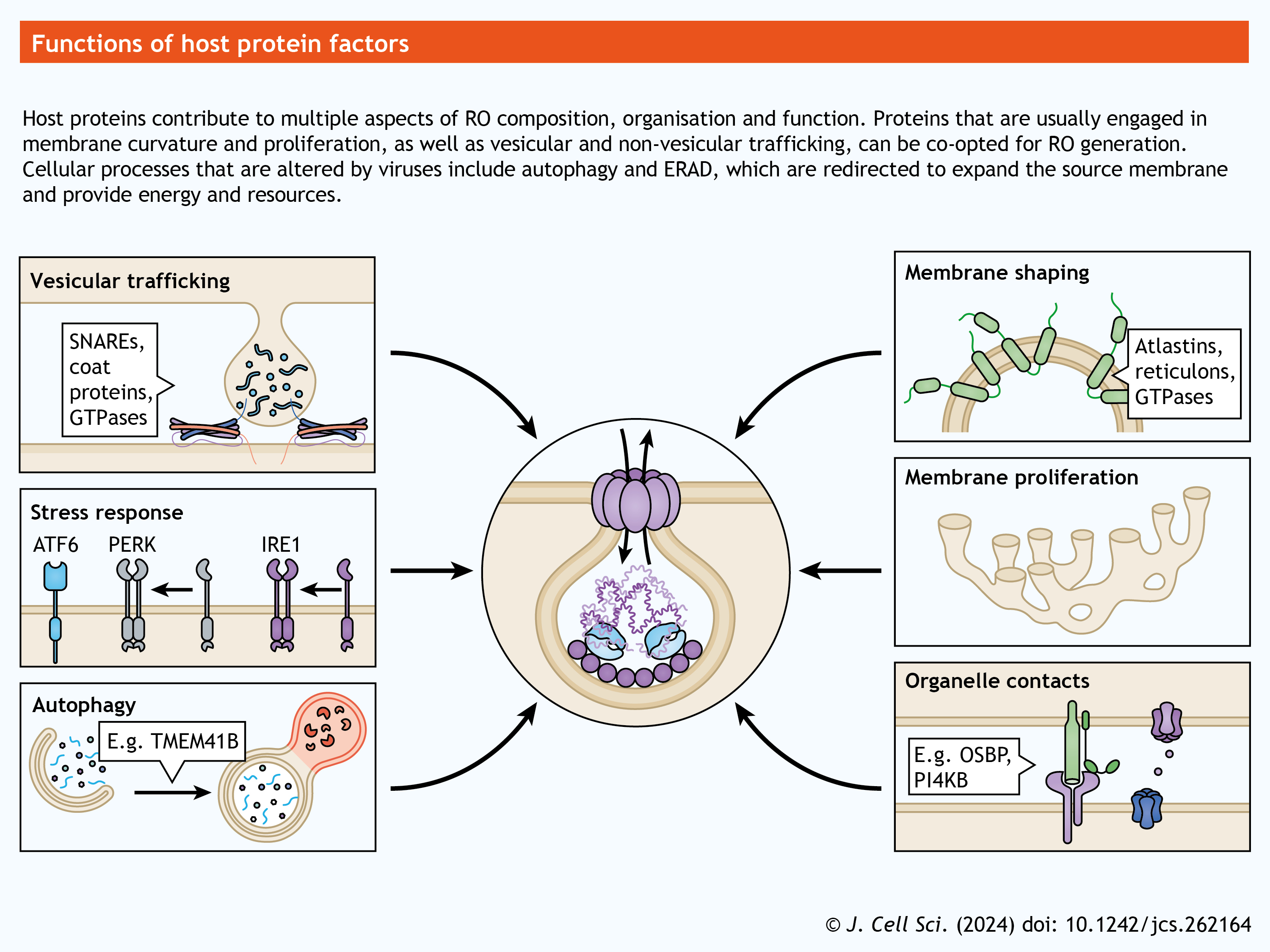

Supplement: Panel 4. Functions of host protein factors [file joces-137-262164-s5.jpg]

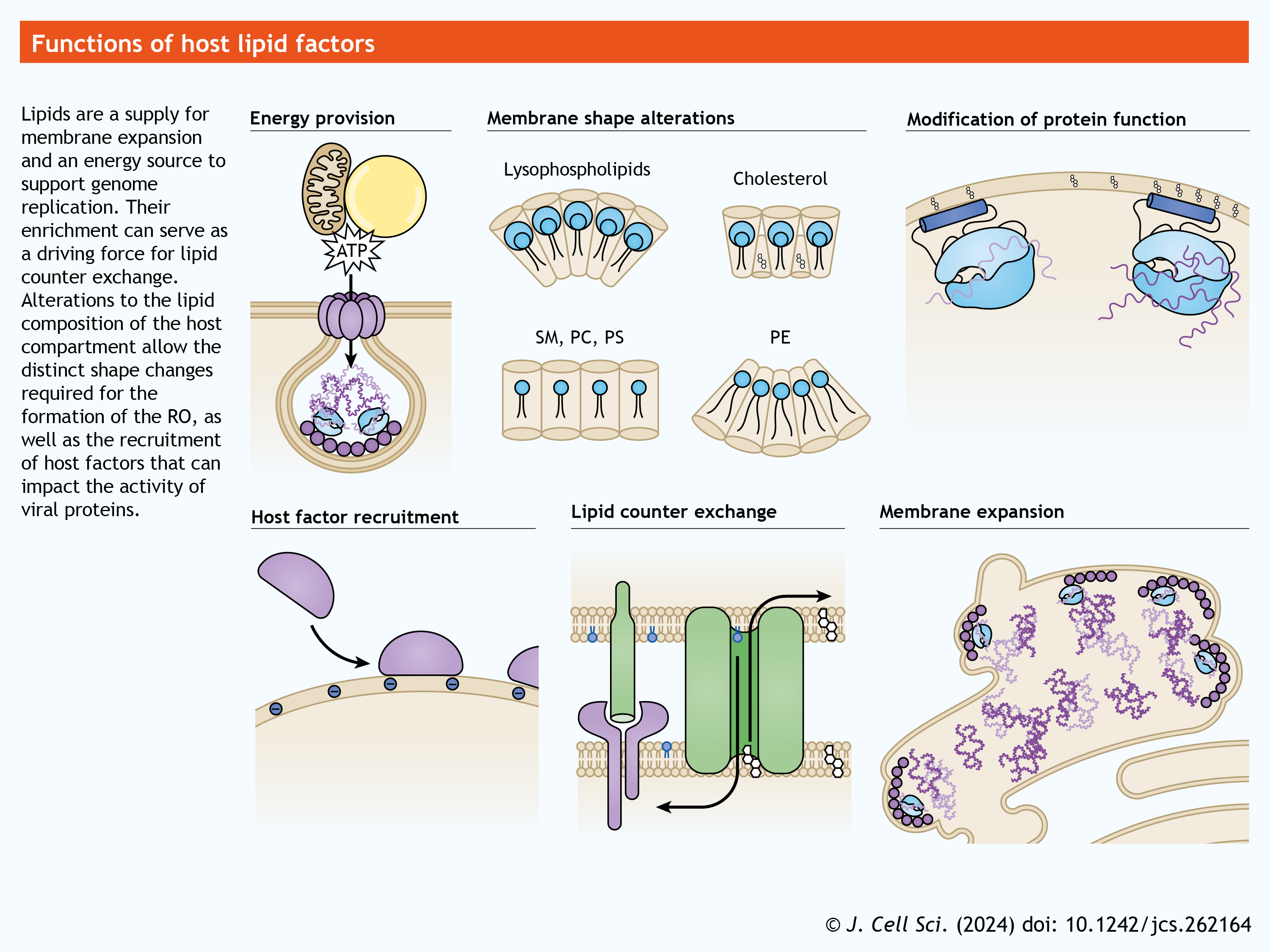

Supplement: Panel 5. Functions of host lipid factors [file joces-137-262164-s6.jpg]

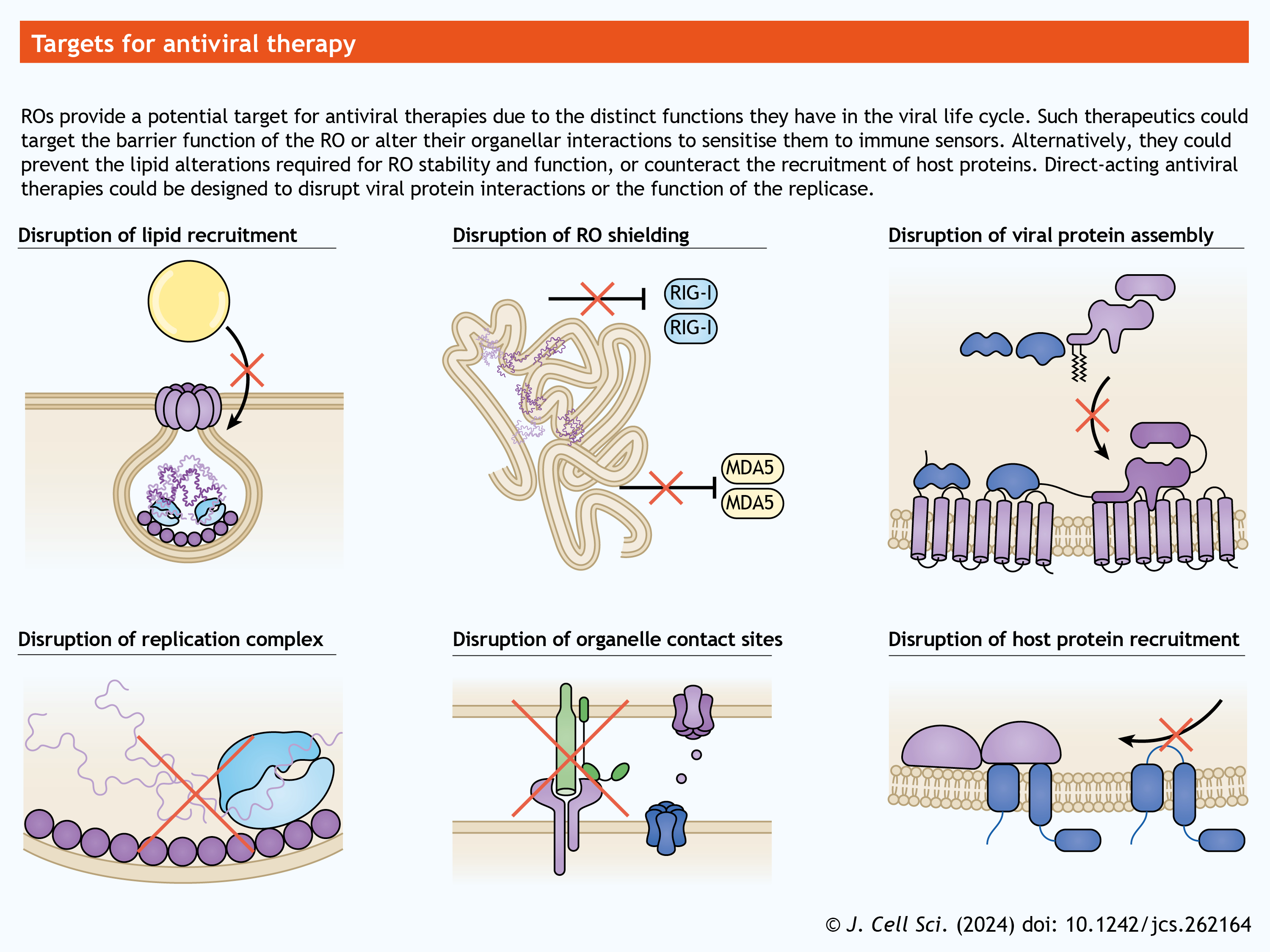

Supplement: Panel 6. Targets for antiviral therapy [file joces-137-262164-s7.jpg]
